# Calorie restriction outperforms bariatric surgery in a murine model of obesity and triple-negative breast cancer

**DOI:** 10.1172/jci.insight.172868

**Published:** 2023-09-12

**Authors:** Kristina K. Camp, Michael F. Coleman, Tori L. McFarlane, Steven S. Doerstling, Subreen A. Khatib, Erika T. Rezeli, Alfor G. Lewis, Alexander J. Pfeil, Laura A. Smith, Laura W. Bowers, Farnaz Fouladi, Weida Gong, Elaine M. Glenny, Joel S. Parker, Ginger L. Milne, Ian M. Carroll, Anthony A. Fodor, Randy J. Seeley, Stephen D. Hursting

**Affiliations:** 1 Department of Nutrition, University of North Carolina at Chapel Hill, Chapel Hill, North Carolina, USA.; 2 Department of Surgery, University of Michigan, Ann Arbor, Michigan, USA.; 3 College of Computing and Informatics, University of North Carolina at Charlotte, Charlotte, North Carolina, USA.; 4 Lineberger Comprehensive Cancer Center, University of North Carolina at Chapel Hill, Chapel Hill, North Carolina, USA.; 5 Department of Pharmacology, Vanderbilt University School of Medicine, Nashville, Tennessee, USA.; 6 Nutrition Research Institute, University of North Carolina at Chapel Hill, Kannapolis, North Carolina, USA.

**Keywords:** Metabolism, Oncology, Breast cancer, Obesity

## Abstract

Obesity promotes triple-negative breast cancer (TNBC), and effective interventions are urgently needed to break the obesity-TNBC link. Epidemiologic studies indicate that bariatric surgery reduces TNBC risk, while evidence is limited or conflicted for weight loss via low-fat diet (LFD) or calorie restriction (CR). Using a murine model of obesity-driven TNBC, we compared the antitumor effects of vertical sleeve gastrectomy (VSG) with LFD, chronic CR, and intermittent CR. Each intervention generated weight and fat loss and suppressed tumor growth relative to obese mice (greatest suppression with CR). VSG and CR regimens exerted both similar and unique effects, as assessed using multiomics approaches, in reversing obesity-associated transcript, epigenetics, secretome, and microbiota changes and restoring antitumor immunity. Thus, in a murine model of TNBC, bariatric surgery and CR each reverse obesity-driven tumor growth via shared and distinct antitumor mechanisms, and CR is superior to VSG in reversing obesity’s procancer effects.

## Introduction

Obesity is an established risk factor for the development and progression of several breast cancer subtypes (1), including triple-negative breast cancer (TNBC), a breast cancer subtype with no targeted therapies and high rates of recurrence (2). The prevalence of obesity in American women now exceeds 40% (3), and effective interventions to lessen the procancer effects of obesity are urgently needed (4–6). Unfortunately, the current literature regarding the reversibility of the obesity–breast cancer link via weight loss interventions is limited. Epidemiologic studies clearly show that bariatric surgery reduces the risk of numerous obesity-associated cancers, including breast cancer (7–10). The most common weight loss surgery in the United States and worldwide is vertical sleeve gastrectomy (VSG) (11, 12). However, all types of bariatric surgery carry surgical complication risks, are very expensive, have strict exclusion criteria, and hence are unavailable to most women with obesity (13). Dietary interventions, which in theory represent widely available and inexpensive means through which weight loss may be achieved, are difficult for many people with obesity to sustain (14–17). Moreover, the epidemiologic literature is limited and inconsistent regarding the ability of dietary weight loss to reverse breast cancer risk or progression in women with obesity (18).

Obesity promotes breast cancer progression through multiple interacting mechanisms, including chronic inflammation. Indeed, obesity alters gene expression and dysregulates systemic adipokines, cytokines, prostaglandins, oxylipins, and other inflammatory markers to promote immunosuppression and procancer signaling in the tumor microenvironment (2, 19). Obesity-associated changes in gut microbial communities appear to contribute to the inflammatory and immune alterations via microbe-derived metabolites (20). Successful, sustained weight loss interventions can modulate many of these mechanisms (5, 21–24), though their causal relationships with reduced breast cancer burden remain unclear (25, 26).

Low-fat diet (LFD) regimens are the most commonly studied weight loss interventions in people with obesity but exhibit consistently poor long-term weight loss success (27). Calorie restriction (CR) is widely effective at reducing obesity-driven tumor incidence and progression in preclinical studies (1, 5) but difficult to implement in people. Intermittent CR (ICR) regimens, where periods of low-calorie consumption are interspersed with unrestricted eating, have emerged as effective weight loss interventions and are more easily implemented than chronic CR (CCR) (28).

Studies in obese animals that characterize the antitumor effects and mechanisms of weight loss achieved via bariatric surgery as compared with calorie restriction interventions (CCR or ICR regimens) have not yet been reported. We partially addressed this gap in a recent report—using the same murine TNBC model and dietary weight loss interventions studied here—and established that the genomic, epigenetic, and antitumor effects of obesity are reversible by CCR and ICR regimens, and, to a lesser extent, LFD (5). Herein, we verify and extend those findings to identify the shared and distinct antitumor effects and mechanisms of VSG relative to LFD, CCR, or ICR in a mouse model of obesity and TNBC. Specifically, we conducted 2 preclinical studies to test the hypotheses that i) weight loss induced by VSG or CR interventions reverses obesity-driven TNBC progression by remodeling the transcriptomic, epigenetic, and immune landscape of tumors and adjacent adipose tissue and ii) weight loss–mediated suppression of tumor growth is concomitant with alteration of intestinal microbiomes, circulating cytokines, and oxylipins.

## Results

### Bariatric surgery and LFD promote weight loss in obese mice, limit obesity-driven TNBC growth, and promote markers of antitumor immunity.

**Figure 1:**
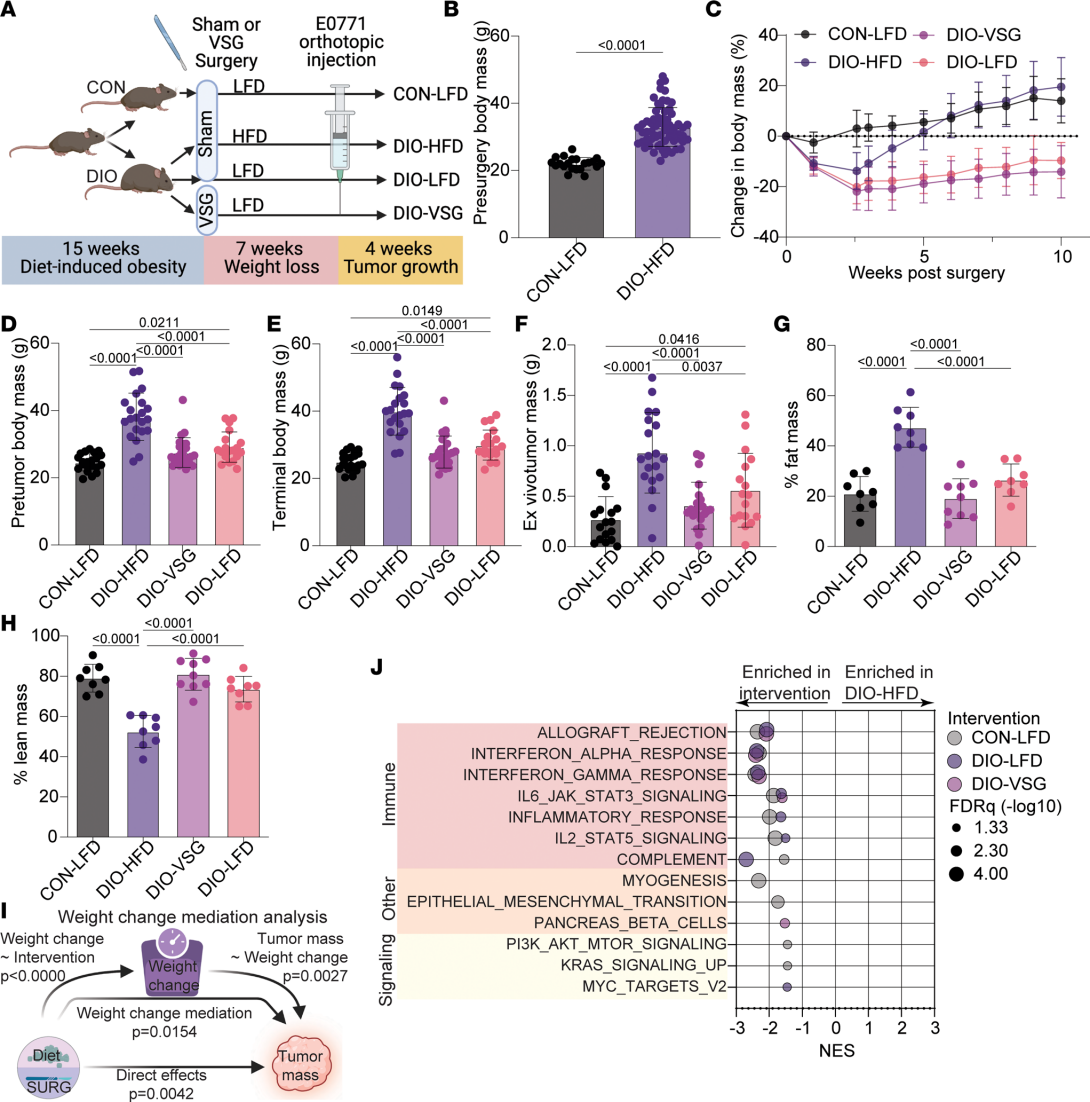
Dietary and surgical weight loss blunt obesity-driven tumor growth. (**A**) Schematic of study design. CON, control; DIO, diet-induced obesity; HFD, high-fat diet; LFD, low-fat diet; VSG, vertical sleeve gastrectomy. (**B**) Body mass prior to weight loss interventions. (**C**) Change in body weight over time following weight-loss interventions. (**D**) Body mass prior to tumor cell injection. (**E**) Terminal body mass. (**F**) Ex vivo tumor mass. (**G** and **H**) Body composition following weight loss interventions. (**I**) Mediation analysis of weight change following weight loss intervention on tumor mass. (**J**) Hallmark gene sets determined significant by GSEA of tumor transcriptomics in pairwise comparisons with DIO-HFD. Gene sets grouped and colored as immune, other, and signaling related. (**B**–**F** and **I**) *n* = 21 CON-LFD, 21 DIO-HFD, 24 DIO-VSG, 19 DIO-LFD. (**G** and **H**) *n* = 8 CON-LFD, 8 DIO-HFD, 9 DIO-VSG, 8 DIO-LFD. (**J**) *n* = 6 CON-LFD, 6 DIO-HFD, 6 DIO-VSG, 5 DIO-LFD. (**B**–**H**) One-way ANOVA with Tukey’s post hoc test. NES, normalized enrichment score.

In study 1 (Figure 1A) we determined whether obesity-driven mammary cancer could be limited by weight loss resulting from VSG or LFD dietary change. Prior to surgery, DIO mice weighed significantly more than CON mice (Figure 1B). While all DIO mice exhibited some weight loss immediately after surgery, only DIO-VSG and DIO-LFD groups sustained this weight loss (Figure 1C and Supplemental Figure 1A; supplemental material available online with this article; https://doi.org/10.1172/jci.insight.172868DS1). Prior to tumor cell injection and at the end of the study, DIO-HFD mice remained heavier than all other groups, and DIO-LFD mice weighed more than CON-LFD mice (Figure 1, D and E). Similarly, ex vivo tumor mass was greater in the DIO-HFD group relative to all other groups (Figure 1F). Tumor mass from DIO-VSG mice was not different from that of CON-LFD or DIO-LFD mice (Figure 1F). Tumor mass was greater in DIO-LFD mice relative to CON-LFD mice (Figure 1F).

We next sought to understand how body composition was altered by VSG and LFD interventions. MRI showed that, relative to the DIO-HFD regimen, each weight loss intervention resulted in reduced relative body fat and increased relative lean mass (Figure 1, G and H). These changes in body composition were largely explained by increased absolute fat mass in DIO-HFD mice relative to all other groups, with no change seen in absolute lean mass (Supplemental Figure 1, B and C). Paralleling this remodeling of adipose tissue, we found that leptin, resistin, glucagon-like peptide-1 (GLP-1), glucagon, TNF-α, and IL-6 were elevated in serum from DIO-HFD mice relative to CON-LFD, and DIO-VSG suppressed this obesity-driven increase (Supplemental Figure 2, A–F). Serum from DIO-LFD mice had a similar overall suppression of obesity-driven elevation of adipokines except glucagon and TNF-α (Supplemental Figure 2, A–F).

Given the similar profile of terminal body weight and tumor mass (Figure 1, E and F) and variation in tumor histology (Supplemental Figure 1D), we used mediation analysis to determine if changes in tumor mass could be explained by proportion of weight lost during the 11 weeks between pre-intervention and study endpoint. We employed mediation analysis to quantify the potential for intervention-driven weight loss to explain terminal tumor mass. Tumor mass was, in part, explained by the percentage of weight lost between weight loss interventions and study end (*P* = 0.0154) and in part by weight change–independent sources of variance (direct effects) (*P* = 0.004) (Figure 1I).

To better understand how these weight loss interventions altered tumor growth, we performed transcriptomic profiling of tumors using Affymetrix microarray, followed by gene set enrichment analysis (GSEA) (29) using Molecular Signatures Database (MSigDB) Hallmark gene sets (30). Relative to tumors from CON-LFD mice, tumors from DIO-HFD mice exhibited suppression of several immune-, differentiation-, and signaling-related gene sets (Figure 1J). Obesity-driven suppression of immune-related gene sets was effectively reversed by both DIO-VSG and DIO-LFD weight loss interventions (Figure 1J). To gain additional insight into the processes altered in tumor transcriptomic profiles, we performed GSEA using Gene Ontology Biological Processes (GOBP) (31) gene sets. To limit redundancy, we subjected all significantly enriched gene sets to enrichment mapping (32) and clustered by similarity coefficient to identify commonly enriched processes and themes. Immune-related signaling dominated the clusters identified for each binary comparison of DIO-HFD with CON-LFD, DIO-VSG, and DIO-LFD (Supplemental Figure 3, A–C).

### Bariatric surgery and dietary weight loss drive distinct transcriptomic changes in mammary adipose tissue.

**Figure 2:**
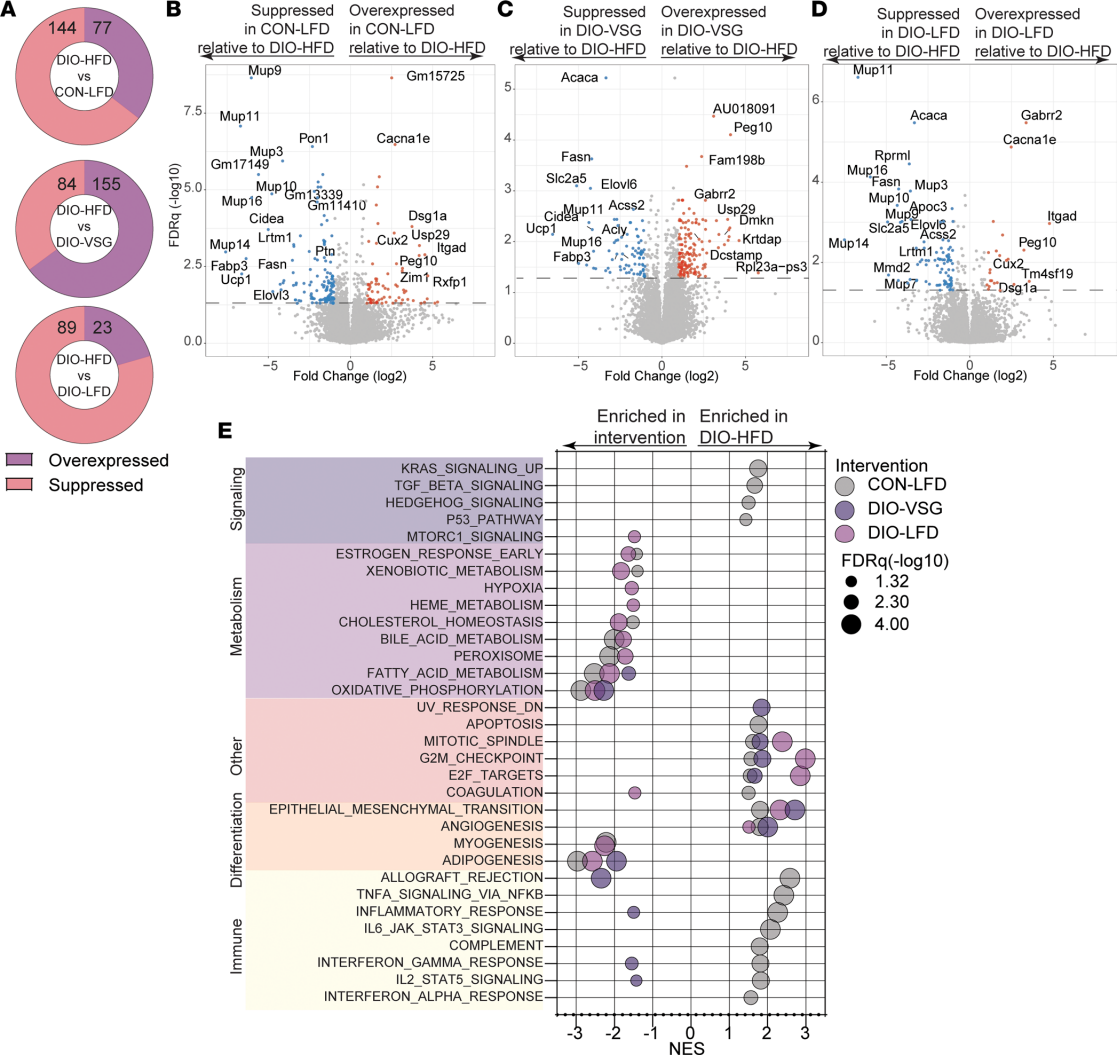
Transcriptomics analysis of mammary adipose tissue following dietary and surgical weight loss reveals discordant metabolic and immune signaling. (**A**) Distribution of differentially expressed genes (DEGs) relative to DIO-HFD. (**B**–**D**) Volcano plots of DEGs generated in the comparison between CON-LFD, DIO-VSG, and DIO-LFD relative to DIO-HFD, respectively. (**E**) Hallmark gene sets determined significant by GSEA of adipose tissue transcriptomics in pairwise comparisons with DIO-HFD. Gene sets grouped and colored as signaling, metabolism, other, differentiation, and immune related. *n* = 4/group.

To understand molecular alterations in adipose tissue gene expression accompanying the changes in body composition, we performed RNA-Seq transcriptomic profiling on mammary adipose tissue contralateral to the tumor. The comparisons of DIO-HFD versus CON-LFD, DIO-HFD versus DIO-VSG, and DIO-HFD versus DIO-LFD revealed numerous differentially expressed genes characterized by a preponderance of overexpressed genes in DIO-VSG relative to DIO-HFD and suppression of gene expression in CON-LFD and DIO-LFD relative to DIO-HFD (Figure 2, A–D).

In contrast to the tumor transcriptomic profiles, GSEA of RNA-Seq data revealed marked enrichment of inflammatory signaling in the mammary tissue from DIO-HFD mice relative to CON-LFD (Figure 2E). Relative to DIO-HFD, weight loss by diet did not alter inflammatory signaling, and VSG only modestly altered inflammatory signaling (Figure 2E). Numerous obesity-driven changes in markers of growth/survival, metabolism, and differentiation were found in the comparison of adipose tissue from DIO-HFD mice with that of CON-LFD mice (Figure 2E). Adipose tissue from DIO-VSG and DIO-LFD mice showed restoration of many of these obesity-driven pathway alterations when compared with adipose tissue from DIO-HFD mice (Figure 2E).

### Bariatric surgery, but not LFD-driven weight loss, drives epigenetic alterations concordant with predicted mediators of transcriptomic changes in adipose from humans who underwent bariatric surgery.

**Figure 3:**
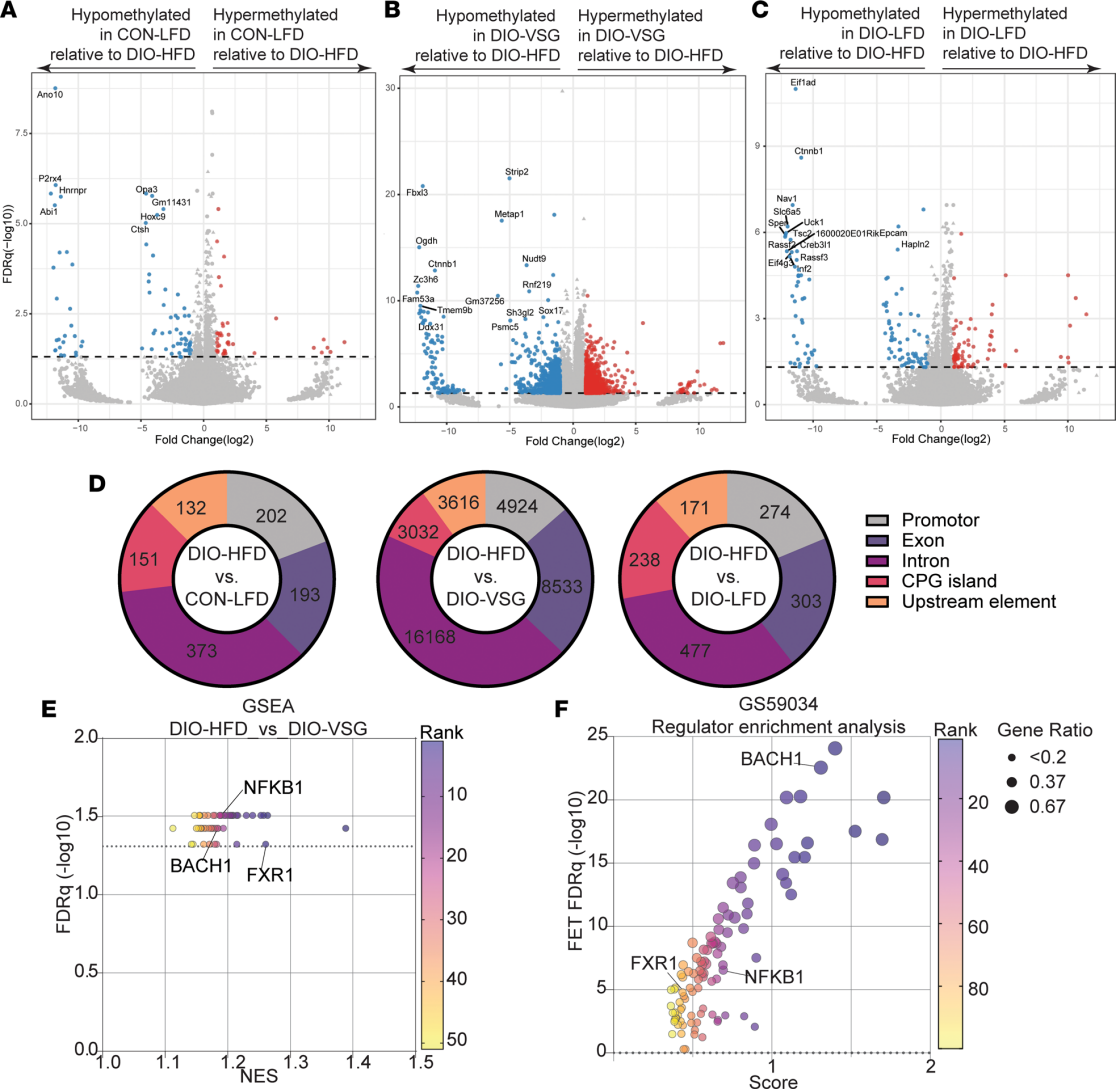
Epigenetic regulation through DNA methylation of mammary adipose tissue reveals transcriptional mediators of the gene expression profile conserved between human and mouse adipose tissue following surgical but not dietary weight loss. (**A**–**C**) Volcano plots of DMGs generated in the comparison between CON-LFD, DIO-VSG, and DIO-LFD relative to DIO-HFD, respectively. (**D**) Distribution of DMGs relative to DIO-HFD. (**E**) MSigDB C3 gene sets determined significant by methylGSA of adipose tissue RRBS in comparison of DIO-VSG with DIO-HFD. (**F**) Regulator enrichment analysis of adipose tissue from patients who were never obese or obese before/after bariatric surgery (GSE59034). (**A**–**E**) *n* = 4/group, (**F**) *n* = 16/group. CPG, cytosine-phosphate-guanine; FET, Fisher’s exact test.

We hypothesized that obesity and weight loss would promote transcriptomic reprogramming in part via changes in epigenetic regulation through DNA methylation. To test this hypothesis, we performed reduced-representation bisulfite sequencing (RRBS) on DNA isolated from mammary adipose tissue contralateral to the tumor. Relative to DIO-HFD adipose tissue, CON-LFD adipose tissue contained 839 differentially methylated genes (DMGs), DIO-LFD adipose tissue contained 1,062 DMGs, and DIO-VSG adipose tissue contained 31,424 DMGs (Figure 3, A–D). To identify transcription factors predicted to regulate these DMGs, GSEA was performed on each pairwise comparison. Only the comparison of DIO-HFD and DIO-VSG demonstrated significant enrichment of transcription factors including NFKB1, BACH1, and FXR1 (Figure 3E). Finally, we sought to determine if the transcription factors that were responsive to DIO-VSG–driven epigenetic changes were also responsive in human adipose tissue following bariatric surgery. We performed RegEnrich analysis of Gene Expression Omnibus (GEO) GSE59034 to identify likely transcriptional mediators of adipose remodeling in humans following bariatric surgery and found all 3 transcription factors (NFKB1, BACH1, FXR1) were significantly associated with gene expression changes following bariatric surgery (Figure 3F).

### Remodeling of fecal microbiotas is associated with weight loss and tumor size.

Using amplicon sequencing of 16S rDNA isolated from fecal matter, we first verified that CON-LFD and DIO-HFD microbiotas were distinct. DIO-HFD microbial communities had fewer observed sequence variants (SVs) relative to the CON-LFD group while Shannon index (which accounts for evenness of microbial composition) was unchanged (Supplemental Figure 4, A and B). β-Diversity was also distinct between CON-LFD and DIO-HFD microbiotas as illustrated by changes in proportional abundances of the 10 most prevalent genera and significant separation of groups by nonmetric multidimensional scaling (NMDS) plot of Bray-Curtis distances (Supplemental Figure 4, C and D). Both weight loss interventions and DIO-HFD reduced the number of observed SVs but not Shannon index relative to CON-LFD–associated microbial communities (Supplemental Figure 4, E and F). The proportional abundances of genera in fecal samples from both weight loss groups were more similar to one another than to other groups (Supplemental Figure 4G). NMDS plot of Bray-Curtis distances revealed close clustering of microbial communities among the weight loss interventions, of which both DIO-VSG– and DIO-LFD–associated communities were different from the fecal microbiota in CON-LFD mice. Further, fecal microbiotas from DIO-LFD were distinct from those from DIO-HFD mice (Supplemental Figure 4H). Finally, we tested the association of each genus with percentage body weight change and terminal tumor mass. While several genera were significantly associated with these percentage body weight changes, no genera were significantly associated with tumor mass (Supplemental Figure 4, I–K).

### Enhanced weight loss through either CCR or ICR more effectively limits obesity-driven TNBC growth than bariatric surgery and restores markers of antitumor immunity.

**Figure 4:**
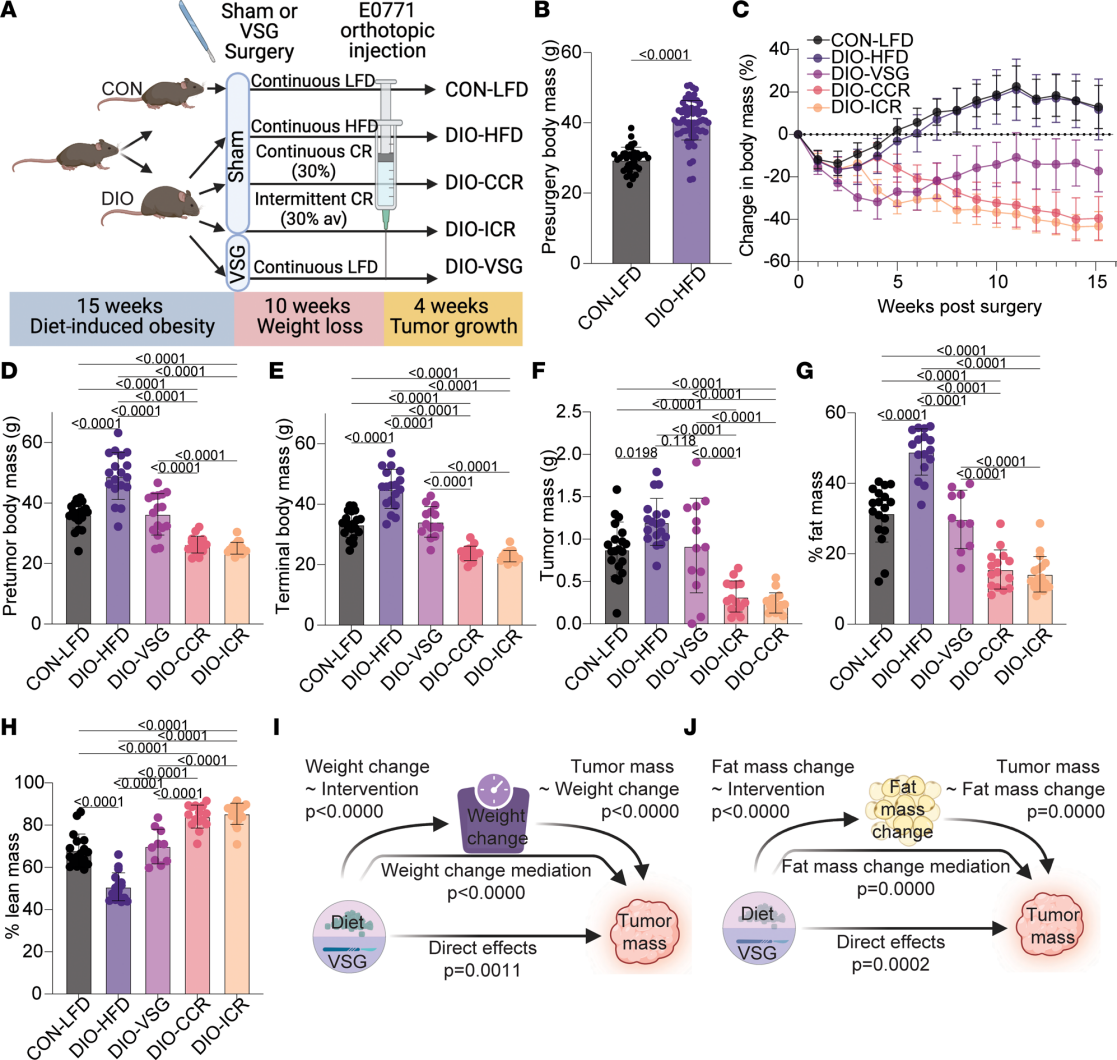
Dietary weight loss via caloric restriction outperforms surgical weight loss to blunt obesity-driven tumor growth. (**A**) Schematic of study design. (**B**) Body mass prior to weight loss interventions. (**C**) Change in body weight over time following weight loss interventions. (**D**) Body mass prior to tumor cell injection. (**E**) Terminal body mass. (**F**) Ex vivo tumor mass. (**G** and **H**) Body composition following weight loss interventions. (**I**) Mediation analysis of weight change following weight loss intervention on tumor mass. (**J**) Mediation analysis of fat mass change following weight loss intervention on tumor mass. *n* = 20 CON-LFD, 18 DIO-HFD, 14 DIO-VSG, 19 DIO-ICR, 16 DIO-CCR. (**B**–**H**) One-way ANOVA with Tukey’s post hoc test.

In study 1 (Figure 1I), weight loss was a significant mediator of smaller tumor size in both the VSG- and LFD-induced weight loss groups and potently remodeled adipose tissue in DIO-VSG mice. Hence, we sought in study 2 (Figure 4A) to determine if greater weight loss achieved through CCR or ICR would mimic or surpass bariatric surgery in limiting mammary cancer growth. As expected, DIO mice weighed significantly more than CON mice prior to weight loss (Figure 4B and Supplemental Figure 5A). While all weight loss interventions promoted significant weight loss, both DIO-CCR and DIO-ICR promoted greater and more sustained weight loss than DIO-VSG (Figure 4, C–E, and Supplemental Figure 5A).

Relative to CON-LFD mice, DIO-HFD mice had significantly larger tumors (Figure 4F). The mean tumor mass of DIO-VSG mice (0.92 ± 0.56 g) was not significantly different from either CON-LFD (0.88 ± 0.32 g) or DIO-HFD (1.20 ± 0.27 g) (Figure 4F). In contrast, mice from both CR interventions — DIO-CCR and DIO-ICR — had significantly smaller tumors relative to all other groups (Figure 4F). At study termination, DIO-HFD mice had a higher proportion of body mass accounted for by fat mass, and a lower proportion by lean mass, than mice from all other groups (Figure 4, G and H). As seen in study 1, increased absolute fat mass, without change in absolute lean mass, in DIO-HFD mice explained differences relative to CON-LFD and DIO-VSG mice (Supplemental Figure 5, B and C). Both DIO-CCR and DIO-ICR mice had reduced absolute fat mass and absolute lean mass relative to other groups (Supplemental Figure 5, B and C). Bariatric surgery changed the composition of lean and fat mass to be not different from CON-LFD mice (Figure 4, G and H). Chronic and intermittent calorie restriction both further reduced percentage fat mass and increased percentage lean mass relative to all other groups (Figure 4, G and H). Similar to study 1, we identified body weight loss (*P* < 0.0001), in addition to weight loss–independent effects (*P* < 0.01), as significant mediators of the antitumor effects resulting from the weight loss interventions in study 2 (Figure 4I). We identified changes in fat mass (*P* < 0.001) and fat mass loss–independent effects (*P* < 0.05) as significant mediators of the antitumor effects of the tested interventions (Figure 4J).

**Figure 5:**
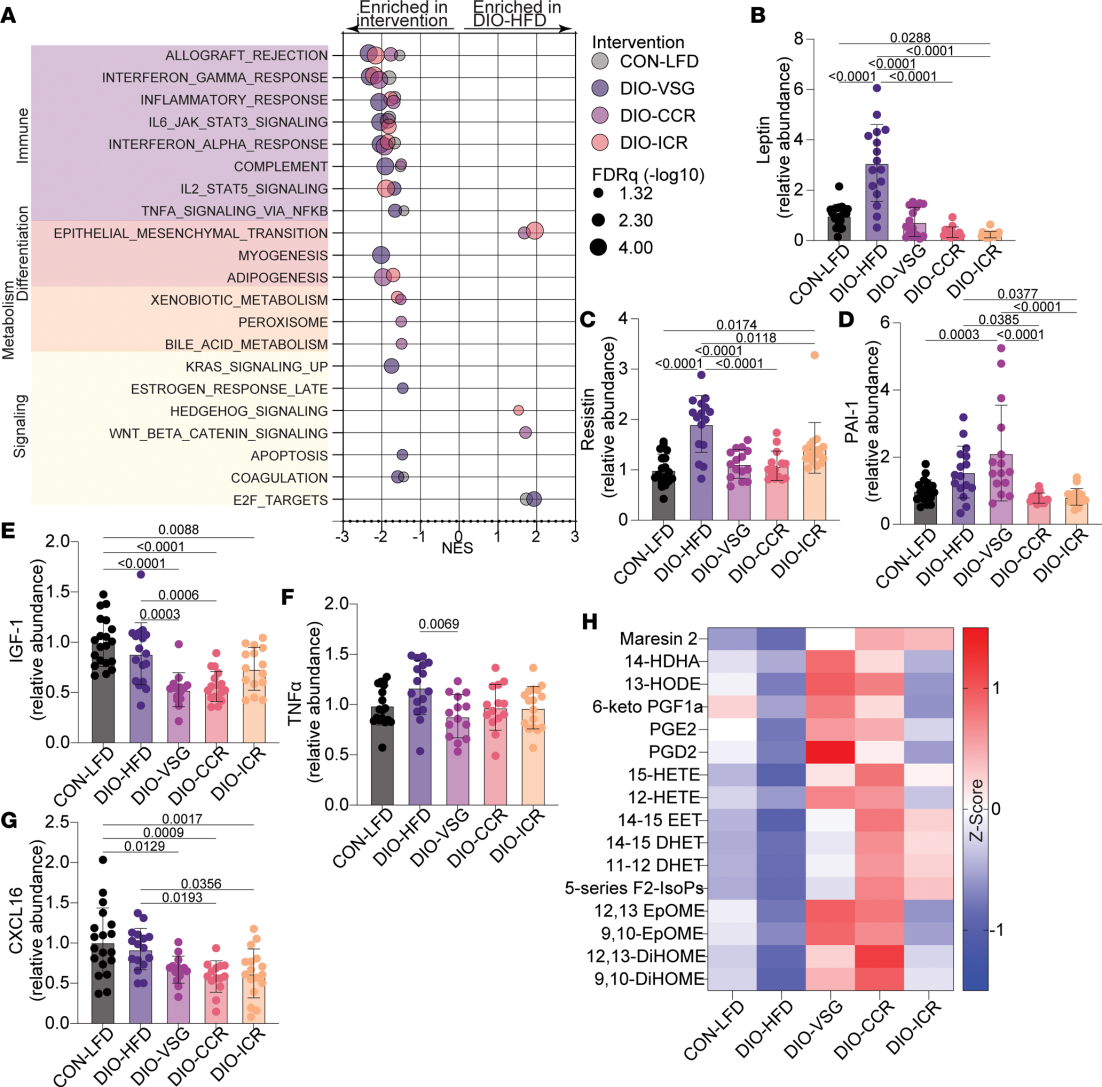
Body weight loss and adiposity mediate blunting of obesity-driven tumor growth by dietary and surgical weight loss interventions. (**A**) Significant GSEA Hallmark gene sets for tumor transcriptomics data in pairwise comparisons with DIO-HFD. (**B**–**G**) Circulating adipokines determined by multiplex ELISA. (**H**) Mammary adipose tissue oxylipin levels determined by UPLC-MS (*z* score). (**A**) *n* = 6/group. (**B**–**G**) *n* = 19 CON-LFD, 16 DIO-HFD, 14 DIO-VSG, 14 DIO-ICR, 17 DIO-CCR. (**H**) *n* = 11 CON-LFD, 10 DIO-HFD, 9 DIO-VSG, 10 DIO-ICR, 13 DIO-CCR.

To understand signaling and pathways altered by the weight loss interventions, we performed tumor transcriptomic profiling followed by GSEA. Concordant with findings from study 1, we identified marked suppression of immune related gene sets in tumors from DIO-HFD mice relative to CON-LFD (Figure 5A). All weight loss interventions (DIO-VSG, DIO-CCR, DIO-ICR) reversed tumoral immunosuppression, as indicated by enrichment of immune-related gene sets relative to DIO-HFD (Figure 5A).

Given the prominent remodeling of obesity-driven adipose tissue dysfunction demonstrated in study 1, we next verified whether similar alterations of circulating adipokines were achieved following either dietary or surgical weight loss in study 2. Serum leptin and resistin levels were increased in DIO-HFD relative to CON-LFD mice (Figure 5, B and C). Both surgical and dietary weight loss reverted obesity-driven elevation of leptin and resistin levels, while only dietary weight loss reverted elevation of plasminogen activator inhibitor 1 (PAI-1) (Figure 5, B–D). Serum insulin-like growth factor 1 (IGF-1) was reduced in DIO-VSG, DIO-CCR, and DIO-ICR mice relative to CON-LFD mice (Figure 5E). Serum CXCL16 was reduced in all weight loss intervention groups relative to the CON-LFD group (Figure 5F). Serum TNF-α was reduced in mice that underwent VSG relative to DIO-HFD mice (Figure 5G). We also found overall suppression of oxylipins in adipose tissue from DIO-HFD mice relative to other groups, particularly DIO-VSG and DIO-CCR (Figure 5H).

### Sustained CR and bariatric surgery promote distinct changes in cecal microbial communities.

**Figure 6:**
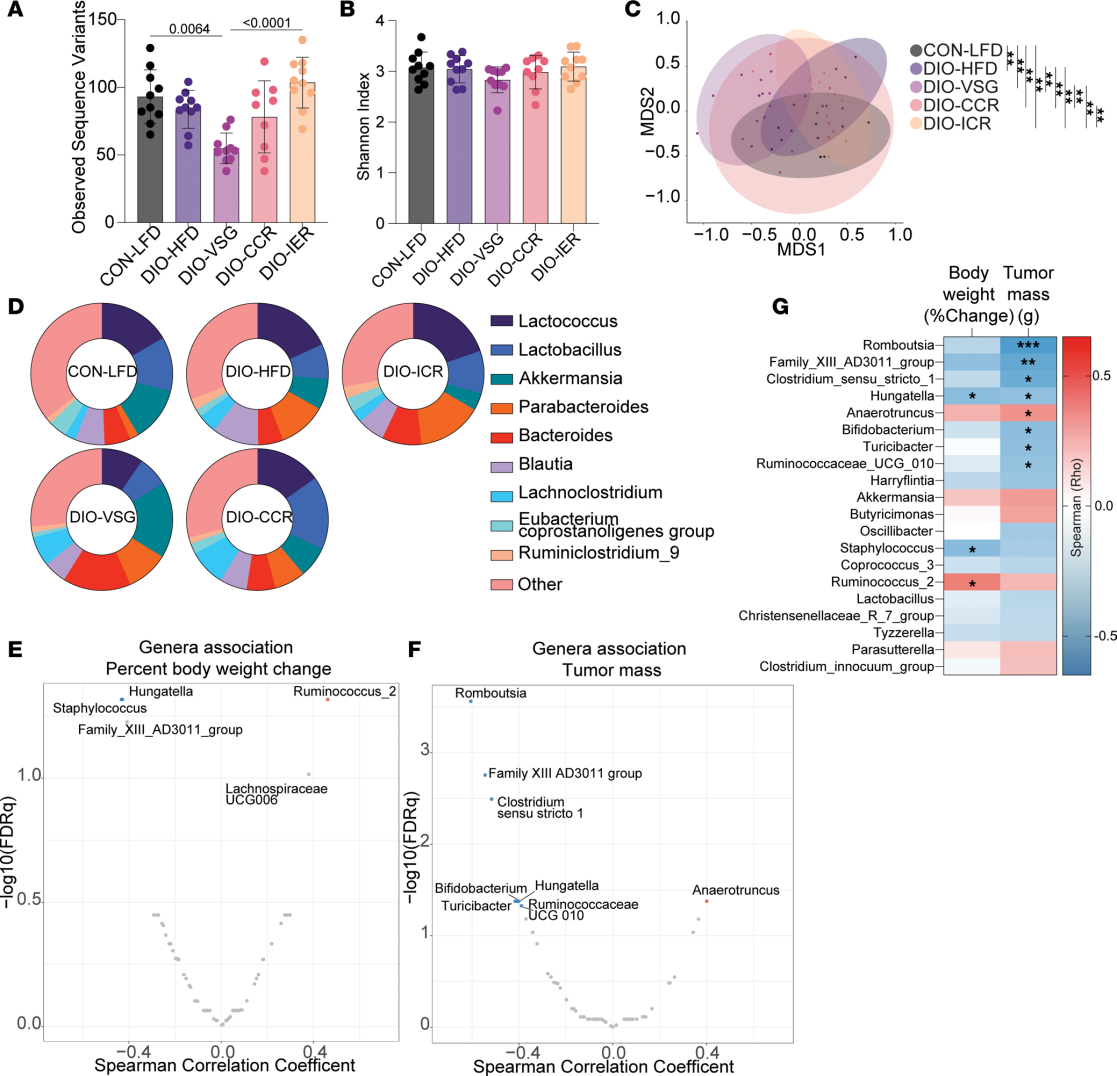
Cecal *Hungatella* abundance associates with both body weight loss and tumor mass. (**A**) Observed SVs. (**B**) Shannon index. (**C**) NMDS plot of Bray-Curtis distances. (**D**) Relative contribution of the 10 most frequent genera to each group. Spearman correlation between all genera and (**E**) percentage body weight change and (**F**) tumor mass. (**G**) Spearman correlation coefficients of the 20 genera showing the highest correlation coefficients with percentage body weight change and tumor mass. *n* = 10/group. *FDRq < 0.05, **FDRq < 0.01, ***FDRq < 0.001.

To assess whether DIO or weight loss in study 2 altered cecal microbial communities, we performed 16S rDNA amplicon sequencing. We found overall limited changes in α-diversity measures of microbial communities across groups as determined by the number of observed SVs and Shannon index (Figure 6, A and B). The β-diversity of cecal microbiotas was significantly different between all intervention groups, illustrated by NMDS plot of Bray-Curtis distances and the taxonomic profiles illustrating the top 10 identified genera (Figure 6, C and D). Additionally, we used Spearman correlation to identify genera significantly associated with percentage body weight change and tumor mass (Figure 6, E–G). Among numerous significant associations, only *Hungatella* was significantly associated with both tumor mass and percentage body weight loss.

## Discussion

Herein we demonstrate that VSG and CR reverse obesity-driven tumor growth via both distinct and shared mechanisms in a murine model of TNBC. Obesity-associated changes in transcripts, epigenetics, secretome, intestinal microbiota, and antitumor immunity all exhibited both redundant and distinct responses to VSG and CR.

We and others have established that obesity-driven immune cell dysfunction drives immunosuppression in the tumor microenvironment that can be reverted by CR regimens (5, 21–24). Further, recent work has demonstrated an association between bariatric surgery and restoration of antitumor immunity (33). We identified shared and unique transcriptional signatures indicative of restoration of antitumor immunity in mice receiving the VSG and CR regimens. These data are congruent with sizable literature supporting an immunomodulatory effect of bariatric surgery in both preclinical and patient-derived samples, principally in adipose tissue and not in the context of breast cancer (4). Indeed, we demonstrate extensive adipose reprogramming following weight loss and identify FXR1, NFKB1, and BACH1 as candidate transcriptional regulators that are common to both human and mouse adipose tissue reprogramming following surgical, but not dietary, weight loss. FXR1, NFKB1, and BACH1 are potent regulators of obesity-driven inflammation and metabolic dysfunction (34–36).

Using mediation analyses, we also found that the extent of total body weight loss or adipose weight loss, regardless of intervention, significantly mediates the reduction in tumor growth (Figure 1I and Figure 4, I and J). This conclusion is supported by substantial literature indicating that obesity-driven adipose inflammation promotes the pro-tumor effects of obesity in models of breast cancer (37).

The intersection of systemic energy balance, intestinal microbiotas, and antitumor immunity is an area of rapid growth, spurred in part by recent findings that the efficacy of immune checkpoint inhibition can be enhanced by fecal matter transplant (38–40). Both dietary and surgical weight loss interventions promote remodeling of intestinal microbiotas (6, 41–43). Here, we found that the abundance of *Hungatella* was associated with both reduced tumor growth and body weight loss. Consistent with our findings, changes in abundance of *Hungatella* species are associated with obesity and body weight and enriched following either dietary or surgical weight loss (44–46). Indeed, increased *Hungatella* in gut microbiomes following antibiotic treatment is associated with nonresponse to immune checkpoint inhibition (47). However, whether regulation of cecal *Hungatella* abundance by obesity is causally related to suppression of antitumor immunity, or potentially to immunotherapy response, is yet to be determined.

Prior work from our group and others shows that in obese mice, weight loss induced by VSG, diet switch to an LFD regimen, or CR is associated with reduced mammary tumor growth (5, 33). Here, we delineate the relative efficacy of each intervention in limiting obesity-driven tumor growth. Further, we identify restoration of antitumor immunity markers and reduction of fat mass as common mechanisms potentially underlying the protective effects of weight loss. Our finding that continuous or intermittent CR had superior efficacy in limiting obesity-driven tumor growth is paralleled in other work showing CR outperforms weight loss driven by ad libitum LFD (5). These findings are particularly germane to the growing interest in the potential for weight loss driven by GLP-1 receptor and/or glucose-dependent insulinotropic polypeptide receptor agonism to limit obesity-driven tumor growth.

This work is limited by the inclusion of only 1 tumor model, albeit implemented across 2 independent studies. We chose to use the orthotopic E0771 transplant model as it is well characterized and widely used in the breast cancer field, and both E0771 tumor growth and immune evasion have been shown to be promoted by obesity (5, 23, 48, 49). The impact of obesity and weight loss interventions in models of other intrinsic molecular subtypes of breast cancer, particularly hormone receptor–positive breast cancer, remains a gap in the field that is not addressed by this work.

Herein we demonstrate that VSG promotes marked remodeling of epigenetic and transcriptional profiles within adipose tissue, with conserved transcriptional mediators across mouse and human data. We find that VSG, CCR, or ICR blunts mammary tumor growth in a weight loss– and adiposity loss–dependent manner. Despite the limitation that only 1 murine model was used, our study comprises comprehensive multiomics analyses of 2 independent study cohorts that inform the complex relationships between weight loss, achieved by bariatric surgery or dietary restriction regimens, and the obesity–breast cancer link. Specifically, in a well-established preclinical model of TNBC, we found that i) CCR, ICR, and to a lesser effect VSG reverse obesity’s procancer effects; ii) shared hallmarks of weight loss–driven reduction of obesity-driven tumor growth in both types of mice include restoration of transcriptomic signatures of antitumor immunity and marked adipose tissue remodeling; and iii) cecal microbiota changes, particularly enrichment in the genera *Hungatella*, are positively associated with both weight loss and reduced tumor growth.

Given the limitations of bariatric surgery, including risk of adverse effects, high cost, and restrictive third-party insurance coverage (33), further translational exploration of efficacious and sustainable dietary weight loss regimens is urgently needed. Our findings in mice orthotopically transplanted with E0771 mammary tumor cells suggest potential avenues for the future development of interventions to limit some of the effects of obesity on TNBC progression.

## Methods

### Animals and diets.

Female 8-week-old C57BL/6NCrl mice were purchased from Charles River Laboratories. Upon arrival, mice were group housed on a 12-hour light/12-hour dark cycle and offered food and water ad libitum. Following 1 week of acclimatization, mice were randomized to receive either an LFD (10% kcal fat; D12450J, Research Diets) or an HFD (60% kcal fat; D12492, Research Diets) to generate normoweight CON or DIO phenotypes, respectively. Body weights were measured weekly. After 15 weeks on diet, CON mice were continued on LFD (CON-LFD), while DIO mice were randomized to continue the same diet (DIO-HFD) or to undergo weight loss via VSG (DIO-VSG) or dietary intervention. In study 1, dietary weight loss was achieved by providing mice ad libitum access to LFD (DIO-LFD). In study 2, dietary weight loss was achieved by providing mice either a 30% CCR diet (DIO-CCR; D15032801 Research Diets) or an ICR diet (DIO-ICR; 14% CR D15032803 and 70% CR D15032804 Research Diets) (5). CCR was accomplished by providing mice 30% fewer daily calories than were consumed by mice in the CON-LFD group. ICR involved providing mice a 14% CR diet for 5 days per week and a 70% CR, high-protein diet on 2 nonconsecutive days per week, thus achieving an average of 30% CR per week relative to the CON-LFD group (5).

### VSG and sham procedures.

VSG and sham procedures were performed in both study 1 and study 2 according to a validated protocol (50). Briefly, VSG involved excision of 70%–80% of the lateral stomach. All mice not undergoing VSG underwent a sham procedure to control for physiological insult of surgery. The sham procedure entailed isolation of the stomach and application of gentle, manual pressure using forceps for 5 seconds. The VSG excision and sham pressure were applied along a line continuous with the esophagus and pylorus. All surgeries occurred within a 5-day window. Preoperative fasting, exposure to isoflurane, and administration of analgesics were consistent across all groups. Mice undergoing VSG were postoperatively provided the same LFD as consumed by the control mice to limit the potential for postoperative aversion to HFDs and to mimic postoperative diet recommendations for patients undergoing VSG (51).

### Tumor model.

Following surgeries, weight loss was closely monitored, and once body weights had stabilized (7 weeks for study 1 and 10 weeks for study 2), all mice were orthotopically injected with 3.5 × 10^4^ E0771 mammary tumor cells into the fourth mammary fat pad (5). In vivo tumor growth was monitored by digital calipers. Mice were euthanized by CO_2_ inhalation 4 weeks following orthotopic injection. Mammary tumors and tumor-adjacent and contralateral mammary fat pads were excised, weighed, and either formalin-fixed and paraffin-embedded or flash-frozen in liquid nitrogen and stored at –80°C until further analysis. Histology was stained with hematoxylin and eosin. Blood was collected by cardiac puncture, allowed to coagulate for 30 minutes at room temperature, and centrifuged at 1,000 rcf for 15 minutes, and then serum was isolated and stored at –80°C. To investigate changes in microbiome composition in tumor-naive mice, fecal samples were collected in study 1 by applying gentle abdominal pressure immediately prior to VSG surgery and again prior to tumor cell injection and were frozen at –20°C. To investigate changes in microbiome composition of a metabolically important site, cecal samples were collected from study 2 at euthanasia, flash-frozen, and stored at –80°C.

### Quantitative MRI analysis.

Quantitative MRI (Echo Medical Systems) was used to measure body composition. Lean body mass, fat body mass, and free water were quantified and expressed as percentage of total weight. A randomly selected subset of 8–9 mice per group were analyzed in study 1, and all mice were analyzed in study 2.

### Nucleic acid extraction.

Total RNA and DNA were extracted from flash-frozen tumor and contralateral mammary fat pad samples using TRIzol Reagent (MilliporeSigma) according to the manufacturer’s instructions. DNA and RNA integrity was determined by TapeStation analysis (Agilent Technologies).

### Tumor transcriptomic analysis by microarray.

Total RNA isolated from tumor tissue was used to synthesize, fragment, and sense-strand label cDNA. cDNA was hybridized to a Mouse Clariom S HT PEG microarray plate (Affymetrix). A GeneTitan MC Instrument (Affymetrix) was used for hybridization, washing, staining, and scanning of the Clariom S peg plate. Data quality control, signal space transformation, and robust multichip average scaling were performed using Transcriptome Analysis Console Software v 4.0.2 (Thermo Fisher Scientific). Published transcriptomic profiling of human adipose samples collected either from patients before or after bariatric surgery or from sex- and weight-matched controls was accessed through GSE59034 and normalized as above.

### Adipose transcriptomic analysis by RNA-Seq.

Total RNA isolated from murine mammary adipose tissue contralateral to the tumor was used to prepare sequencing libraries with Illumina TruSeq Stranded Total RNA Sample Preparation kit and sequenced using an Illumina HiSeq 2000 instrument. FASTQ files were aligned to the mm10 mouse genome (GRCm38.p4) using STAR v2.4.2 (52) with the following parameters: --outSAMtype BAM Unsorted --quantMode TranscriptomeSAM. Transcript abundance for each sample was estimated with salmon v0.1.19 (53) to quantify the transcriptome defined by Gencode gene annotation. Gene level counts were summed across isoforms, and genes with low expression (defined as fewer than 10 counts across all samples) were removed before downstream analyses. DESeq2 (54) was used to test for differentially expressed genes between interventions.

### GSEA.

GSEA (v4.3.2) was conducted to identify pathways and processes transcriptionally altered by our interventions in both adipose tissue and tumor (29, 30) analysis using SST-RMA–normalized microarray data and DESeq2–normalized (54) RNA-Seq data. Enrichments were calculated for GSEA Hallmarks and GOBPs. Enrichment mapping was performed to cluster significant (FDR *q* < 0.05) gene sets by similarity index of genes and to limit redundancy across significant GOBP GSEA results (32).

### DNA methylation analysis by RRBS.

Genome-wide methylation profiles for the mammary fat pad contralateral to the tumor were determined by RRBS. Library preparation and sequencing were performed at the University of North Carolina at Chapel Hill High-Throughput Sequencing Facility. Alignment and differential methylation analysis were conducted as previously described (55). FASTQ files were aligned to the mm10 mouse genome using Bismark v0.18.1 with default settings (56). BAM files were then sorted with samtools v1.5 (57), and methylation calls were generated using methylKit R package v1.10.0 (58). Bases with low variability (standard deviation of methylation level < 0.05) and extreme read coverage (>5,000 times summed across all samples) were removed to avoid PCR artifacts. Data were visualized using NMDS plots. Two outliers (1 CON-LFD, 1 DIO-LFD) were removed from the downstream analysis due to their poor sequencing quality. For RRBS data, methylGSA R package v1.2.3 (59) was used to test for significantly enriched gene sets.

### Adipose tissue oxylipin analysis.

Mammary adipose tissue contralateral to the tumor was homogenized, and lipids were extracted in methanol, centrifuged at 15,000 rcf for 3 minutes, and loaded onto an Oasis MAX micro-elution plate (Waters Corp). Oxylipins were washed in methanol and eluted using propanol/acetonitrile (50/50, v/v) containing 5% formic acid. Oxylipins were separated using a Waters Corp. Acquity I-Class UPLC and detected using a Waters Corp. Xevo TQ-XS triple-quadrupole mass spectrometer operating using multiple reaction monitoring in negative ion mode with argon as the collision gas. Oxylipins were quantified using the ratio of sample signal to internal isotopically labeled standard peak height and normalized to mass of adipose tissue used.

### 16S rDNA amplicon sequencing.

DNA was extracted from fecal and cecal samples following 40 minutes of vortexing in 0.5 mL of QIAGEN PM1 buffer with 200 mg of 106/500 μm glass beads (MilliporeSigma). A KingFisher Flex Purification System was used with QIAGEN ClearMag beads to purify DNA (University of North Carolina [UNC] Microbiome Core). 16S 515–806 bp (variable region 4) was amplified from DNA extracted from cecal contents, and 16S 27–338 bp (variable region 1–2) was amplified from DNA extracted from fecal samples. PCR amplicons were then sequenced using an Illumina MiSeq platform (UNC High-Throughput Sequencing Facility). SVs were identified using the DADA2 pipeline (60), with taxonomic classification performed using DADA2 with the SILVA reference database (61). Samples with fewer than 10^4^ total aligned reads and SVs with a frequency less than 0.01% were removed prior to analysis. Reads were subjected to total sum scaling. The α-diversity of 16S amplicon sequences was expressed as Shannon index and total observed SV count using Microbiome Analyst (62). The β-diversity was assessed using NMDS plots of Bray-Curtis distances calculated using the vegan package (63), and pairwise PERMANOVAs were performed using the pairwiseAdonis package. Association of all genera, with percentage body weight change and tumor mass was performed by Spearman correlation in R, subject to multiple-hypothesis correction.

### Serum secretome analyses.

Serum hormone, cytokine, and adipokine concentrations were measured using a Milliplex Mouse Metabolic Hormone Magnetic Bead Panel (MMHMAG-44K), a Bio-Plex Pro Mouse Adiponectin Assay, and a 6-Plex Mouse Cytokine Panel (Bio-Rad Laboratories). IGF-1 concentrations were measured using an R&D Systems IGF-1 Bead-Based Single-plex Luminex assay.

### Statistics.

One-way ANOVA followed by Tukey’s post hoc test was used to assess the effects of diet and weight loss across groups. For α-diversity measures Kruskal-Wallis or Mann-Whitney *U* tests were used. PERMANOVA was used to assess average Bray-Curtis distances between groups. Correction for multiple testing was achieved using the Benjamini-Hochberg procedure for all transcriptomic, epigenetic, and 16S rDNA amplicon sequencing. Results were analyzed using GraphPad Prism software and R version 3.4.3. *P* ≤ 0.05 was considered statistically significant. All means are presented with error bars indicating standard deviation.

### Study approval.

All animal study protocols were approved by and coordinated in compliance with guidelines issued by the University of North Carolina at Chapel Hill Institutional Animal Care and Use Committee.

### Data availability.

Microarray, RNA-Seq, and RRBS data sets have been deposited in the NCBI GEO under the accession number GSE230474. Individual values for all other data are available online in the Supporting Data Values XLS file.

## Author contributions

MFC, KKC, and SDH conceived the study. MFC, KKC, AGL, FF, WG, JSP, RJS, and AAF developed methodology. MFC, KKC, TLM, SSD, FF, WG, JSP, and AAF developed software. MFC and KKC validated data. MFC, KKC, TLM, SSD, FF, WG, JSP, and AAF performed formal analysis. MFC, KKC, TLM, SSD, ETR, AGL, SAK, AJP, LAS, LWB, EMG, and GLM investigated. MFC, KKC, WG, and EMG curated data. MFC, KKC, EMG, and SDH wrote the original draft. MFC, KKC, LWB, FF, EMG, JSP, IMC, RJS, and SDH reviewed and edited the draft. MFC and SSD visualized data. JSP, RJS, AAF, and SDH supervised. SDH acquired funding. MFC and KKC made critical contributions to this work and merit sharing first authorship. They are listed in alphabetical order.

## Supplementary Material

Supplemental data

Supporting data values

## Version 1 09/12/2023

In-Press Preview

## Version 2 10/09/2023

Electronic publication
